# Factors driving increases in *Clostridioides difficile* infection rates in England: a national case−control study, 2019 to 2024

**DOI:** 10.2807/1560-7917.ES.2026.31.37.2500888

**Published:** 2026-09-17

**Authors:** Tim Whiteley, Andre Charlett, Dimple Chudasama, Zahin Amin-Chowdhury, Russell Hope, Laura Fellowes, Dakshika Jeyaratnam, Rebecca Oettle, Julie V Robotham, Charlotte Stevens, James Stimson, Thomas Inns, Stephanie Evans

**Affiliations:** 1 UK Health Security Agency, Colindale, London, United Kingdom

**Keywords:** *Clostridioides difficile*, CDI, logistic regression, antibiotic prescribing

## Abstract

**BACKGROUND:**

Numbers of *Clostridioides difficile* infections (CDI) in England have risen since 2021, but for unclear reasons.

**AIM:**

This study’s objective was to understand factors driving CDI increases, and their relative impact.

**METHODS:**

We conducted a case–control study in England from 2019/20 to 2023/24, with CDI case data linked to hospitalisation episodes. Controls were non-CDI related hospitalisation episodes. A logistic regression model estimated odds ratios (ORs) for primary care prescribing, comorbidities, demographic factors, region and testing rates. To assess temporal changes, the model was fitted to 2019/20 data to predict, from 2020/21 onwards, case numbers, which were compared to observations. The relative contribution of factors to the model performance was evaluated by their stepwise inclusion into it, starting from a baseline considering hospitalisation numbers. Prediction improvements were assessed after each incorporation.

**RESULTS:**

The strongest CDI risk factors were prescriptions (e.g. OR for clindamycin: 5.22; p < 0.001), comorbidities (OR: 1.17; p < 0.001), and age (OR: 1.03; p < 0.001). All ethnicities except White negatively associated with CDIs. Among regions, the North West and Yorkshire and Humber had the highest ORs (1.17 and 1.15 respectively; p < 0.001). Demographic changes and antibiotic use explained 12.6% of the difference between observed cases in 2023/24 and our baseline model whereas including comorbidities and testing rates explained 65.3%.

**CONCLUSION:**

Increases in overall hospital admissions, comorbidities and testing largely explained rising case numbers, which also stemmed from an ageing population, demographic changes and more primary care antibiotic prescribing**.** Nevertheless, these factors did not fully account for observed increases, so further determinants should be sought.

**Key public health message**

**What did you want to address in this study and why?**

*Clostridioides difficile* infection (CDI) is a major healthcare-associated bacterial infection that has a devastating impact on patients. In England, the rates of CDIs have been rising since 2021 after a decade of stability. This study aimed to pinpoint key factors behind CDI increases and investigate whether they were sufficient to explain the observed situation evolution.

**What have we learnt from this study?**

The key risk factors behind CDIs are antibiotic prescribing, comorbidities and age. Our model predicted a trend of increasing cases, which was similar to that of observed case. The main explanations for the rise in hospitalisations were comorbidities and testing. However, from 2020/21 onwards, our model predicted fewer cases than we actually saw, implying there is one or several key factors behind rises in CDI cases that we did not include in our model.

**What are the implications of your findings for public health?**

Practitioners should be aware of the risks associated with CDI, especially the risks of antibiotic prescribing on individuals. Hospital staff should be aware of the explainable rise in cases and prepare for future years accordingly. Practitioners should explore the hospital-level reasons behind the unexplained case rise and ensure that the highest levels of infection prevention and control are maintained.

## Introduction

*Clostridioides difficile* infection (CDI) is a major healthcare-associated infection worldwide linked to increased risk of morbidity and mortality [1]. The causative agent is a spore-forming, toxin-producing bacterium that induces a spectrum of clinical manifestations ranging from mild diarrhoea to severe colitis, which may be life-threatening, particularly in older adults and individuals with underlying comorbidities [1-4]. Spores of *C. difficile* are highly resistant to heat and many commonly used disinfectants, enabling prolonged environmental persistence, and from where they can be cross-transmitted to patients, typically through the faecal–oral route [5].

There is a considerable economic healthcare burden associated with CDI, with hospitalisation of an initial CDI episode requiring an average length of stay of 17 days and potentially extending to 33 days for recurrent infection [1]. A study in 2018 estimated that average total costs per patient to a National Health Service (NHS) hospital ranged from GBP 12,710 (EUR 14,695) to GBP 31,121 (EUR 35,983) [1].

Risk factors for CDI include older age (≥ 65 years), recent antibiotic use, the number of antibiotics prescribed and prior hospitalisation [6]. Recurrent CDI infections are common, with around a quarter of patients experiencing a recurrence, of which as many as 40–60% multiple recurrences [7]. The consequence of CDI on patients’ health-related quality of life is devastating with an emotional impact that continues after infection, resulting in isolation and fear of recurrence [8].

In 2018/19, CDI rates in England were at their lowest level since surveillance began, at 21.9 cases per 100,000 – declining from 107.6 cases per 100,000 at start of surveillance in 2007/08 [9]. These reductions followed the rollout of several nationally targeted efforts to drive CDI rates down, which emphasised five key areas associated with CDI: prudent antibiotic prescribing, hand hygiene, environmental decontamination, isolation/cohort nursing and personal protective equipment (PPE) [10]. This also corresponded with the decline of the fluoroquinolone-resistant 027 strain [9,11]. Subsequently, rates remained broadly stable between 2013/14 until 2020/21 (ranging from 21.9 to 26.1 cases per 100,000 people). From 2020/21, the rates of CDI began increasing annually with a 33% increase in cases between 2020/21 and 2023/24 seen nationally. Increases occurred across all age groups and sexes, and among both hospital- (HO) and community-onset (CO) cases. A descriptive analysis of this rise in cases identified changes in multiple factors which were known to be associated with CDI but could nevertheless not identify a clear reason for it [9]. In particular, analysis of ribotyping data from the *Clostridioides difficile* Ribotyping Network (CDRN), did not indicate a particular clonal expansion behind the CDI increases observed [9,12,13].

Internationally, the United Kingdom (UK) has middling to lower CDI case rates per head of population though comparisons are difficult due to differences in definitions and detection methods [9,14,15]. The trend of global cases has generally been one of decreasing prevalence since peaks in the late 2000s and current rates appear stable with fluctuations shown by some countries but no clear global epidemiological change [14,15].

In England, comprehensive understanding of the epidemiological trend and risk factors driving the increase in CDI is essential for informing targeted interventions and policy decisions. The aim of this study was to understand the risk factors associated with CDI and what proportion of the recent increase could be explained by changes to these risk factors.

## Methods

### Study design

We undertook a population-level case–control study using a logistic regression to estimate the likelihood of an individual acquiring CDI given they have a hospitalisation episode. The study population was all individuals with a hospital admission to any NHS acute trust in England between 01 April 2019 and 31 March 2024 and recorded in the Hospital Episode Statistics (HES) database, which hosts data on a total of 82 million admissions. Patients living outside England but admitted to trusts within England were not excluded.

The study cases were defined as individuals within the study population for which there was a laboratory-confirmed CDI reported to the UK Health Security Agency (UKHSA)’s mandatory surveillance system [16] during the study period (n = 71,052). There was no constraint applied to whether the CDI occurred before, during or after the hospital stay for which there was an HES record. The hospital admission used to provide information on patient characteristics was not necessarily associated with the reported CDI.

The study cases were categorised as CO CDI cases (patients who tested positive and were treated for CDI in the community and patients who were already infected on admission to hospital) and as HO CDI cases (patients who were infected during their hospital stay) [17]. For modelling simplicity, CDI case categorisation by prior healthcare exposure was not used in this study. The effect of previous hospitalisation was considered using a separate exposure variable.

Controls were defined as the remaining admissions during the study period which were unlinked to a CDI case. To manage data size, a random 1% sample of controls was selected. In Supplement 1 we show that the model is stable even if a smaller sample size was used. Cases and controls were not matched on any characteristic.

We defined the key date of CDI as the date of positive test result for cases and the date of hospitalisation for controls. Variables included in the model were: age; sex (included as a binary variable, as further described in Supplement 2); ethnicity; UKHSA region; index of multiple deprivation (IMD) from the patient’s home lower super output area (LSOA); frailty (Dr Foster fragility metric from Soong et al. [18]); cancer; renal disease; number of comorbidities (excluding cancer and renal); previous nights in hospital over the last 12 months; tests per 1,000 admissions at their trust; individual level primary care antibiotics and proton pump inhibitor (PPI) prescribing in the 6 months preceding the key date; financial year (spanning 1 April−31 March) and quarter. Details on the datasets used, reference populations and variable coding specifications can be seen in Supplement 2. Details on the sensitivity analysis to justify the choice of 6 months for antibiotics can be seen in Supplement 3.

### Analysis

Initial investigations into missingness within the data showed that several hospital trusts had significant biased missingness for trust testing rate and patient sex. We removed data relating to 38 trusts – 36 with an incomplete CDI testing rate history for the reporting period; and two with missing data on patient sex. Moreover, patient age, sex and hospitalisation records were also cleaned. Overall, we excluded 28.1% of cases (19,979/71,052) and 33.3% (271,736/815,327) of controls, retaining 51,073 cases and 543,591 controls. Details can be seen in Supplement 2.

The analysis to produce our logistic regression model was performed in R 4.4.0 [19] using the generalised linear model (glm) function which is used to fit glms. The output metrics of interest were odds ratios (ORs) and total predicted case numbers. Control cases used *weights = 100* to rebalance, given that we used a 1% sample of our total controls. Using this weighted sampling strategy, we can estimate both ORs and the probability of infection given hospitalisation. Univariable and multivariable analyses were performed using the same methodology. Code is available at https://github.com/ukhsa-collaboration/CDI_LR_external although the data to run this model is sensitive and therefore not publicly available.

In the first part of the analysis, we fitted this model to all data from 2019/20 to 2023/24 and identified CDI risk factors in the dataset. To focus on HO, we excluded the CO case data before fitting the model and estimating ORs (and vice versa to get CO cases). This gives the ORs showing the factors that impact on CDI.

In the second part, we sought to understand what has changed between 2019 and 2024 that might cause an increase in cases. We used financial year 2019/20 data only to fit the model coefficients (without financial quarter) and then predicted cases in future years and compared them to the number of observed cases.

To understand the contribution of each factor to the model prediction, we broke this full model into sub-models of increasing complexity. We started by fitting a baseline model of hospitalisations only (*cdi*~1) to 2019/20 and predicting forward. Then we added a group of variables to the equation, fitted the new model to 2019/20 and predicted forwards. We continued adding groups of variables, beginning with those perceived to be more distal and then those that are more proximal until we reached the full model (the original model without financial quarter). This gave us the following:

- Baseline (*cdi*~1), which accounts for **total admission numbers** only;

- Model 1 accounting for **demography**, including age, sex, ethnicity, IMD and region;

- Model 2 as above plus **prescribing**;

- Model 3 as above plus **comorbidities** and previous hospitalisation;

- The full model as above plus **testing**.

In this part of the analysis, financial year and quarter were not included as variables in any of the models.

## Results

### Risk factors for *Clostridium difficile* infection

Table 1 shows the breakdown for cases and controls for each variable. Compared with controls, the cases were older (mean 71.5 years compared with 53.4), predominantly of white ethnicity (91.6% compared with 84.5%), frailer (mean 1.9 compared with 0.51), more frequently with renal disease (26.4% compared with 12.4%) and appeared more likely to have had antibiotics (e.g. 11.3% compared with 3.5% recorded as having had amoxicillin–clavulanic acid). Moreover, cases had a mean of 26.7 previous nights in hospital in the preceding 12 months, compared with 4.2 for the controls.

**Table 1:** Characteristics of the cases and controls used in the model, England, 2019–2024 (n = 594,664)

Characteristics		Controls (n = 543,591)	Cases (n = 51,073)
Age mean in years (SD)		53.37 (25.18)	71.46 (18.31)
Sex (%)	Female	296,164 (54.5)	29,163 (57.1)
	Male	247,427 (45.5)	21,910 (42.9)
Ethnicity (%)	White	459,437 (84.5)	46,764 (91.6)
	Asian	38,702 (7.1)	1,729 (3.4)
	Black	18,708 (3.4)	682 (1.3)
	Mixed	8,105 (1.5)	223 (0.4)
	Other	3,883 (0.7)	134 (0.3)
	Unknown	14,756 (2.7)	1,541 (3.0)
Financial year and quarter (%)	2019/20 Q1	28,792 (5.3)	2,140 (4.2)
	2019/20 Q2	29,195 (5.4)	2,540 (5.0)
	2019/20 Q3	29,480 (5.4)	2,421 (4.7)
	2019/20 Q4	27,044 (5.0)	2,172 (4.3)
	2020/21 Q1	16,548 (3.0)	1,931 (3.8)
	2020/21 Q2	22,987 (4.2)	2,414 (4.7)
	2020/21 Q3	24,543 (4.5)	2,289 (4.5)
	2020/21 Q4	22,472 (4.1)	2,137 (4.2)
	2021/22 Q1	26,935 (5.0)	2,510 (4.9)
	2021/22 Q2	27,238 (5.0)	2,844 (5.6)
	2021/22 Q3	27,282 (5.0)	2,592 (5.1)
	2021/22 Q4	26,796 (4.9)	2,319 (4.5)
	2022/23 Q1	27,583 (5.1)	2,694 (5.3)
	2022/23 Q2	28,091 (5.2)	3,135 (6.1)
	2022/23 Q3	28,678 (5.3)	2,622 (5.1)
	2022/23 Q4	28,959 (5.3)	2,561 (5.0)
	2023/24 Q1	29,142 (5.4)	2,825 (5.5)
	2023/24 Q2	29,957 (5.5)	3,032 (5.9)
	2023/24 Q3	30,632 (5.6)	2,920 (5.7)
	2023/24 Q4	31,237 (5.7)	2,975 (5.8)
Frailty mean (SD)		0.51 (1.25)	1.90 (2.46)
Renal disease (%)	False	476,271 (87.6)	37,588 (73.6)
	True	67,320 (12.4)	13,485 (26.4)
Previous CIPS duration mean nights (SD)		4.24 (19.54)	26.66 (43.21)
Cancer (%)	False	466,401 (85.8)	42,311 (82.8)
	True	77,190 (14.2)	8,762 (17.2)
Comorbidities mean number (SD)^a^		0.60 (0.91)	1.31 (1.23)
Testing rate mean (SD)		40.67 (19.25)	44.28 (20.40)
IMD (%)	1	46,659 (8.6)	4,419 (8.7)
	2	55,181 (10.2)	5,344 (10.5)
	3	52,781 (9.7)	4,770 (9.3)
	4	50,679 (9.3)	4,661 (9.1)
	5	50,915 (9.4)	4,814 (9.4)
	6	51,433 (9.5)	4,968 (9.7)
	7	50,980 (9.4)	4,960 (9.7)
	8	50,707 (9.3)	4,851 (9.5)
	9	49,533 (9.1)	4,965 (9.7)
	10	48,924 (9.0)	4,838 (9.5)
	Unknown	35,799 (6.6)	2,483 (4.9)
Region (%)	East Midlands	41,702 (7.7)	4,009 (7.8)
	East of England	46,720 (8.6)	4,458 (8.7)
	London	80,977 (14.9)	4,888 (9.6)
	North East	36,201 (6.7)	3,543 (6.9)
	North West	74,516 (13.7)	9,345 (18.3)
	South East	84,890 (15.6)	7,912 (15.5)
	South West	58,155 (10.7)	5,926 (11.6)
	West Midlands	55,816 (10.3)	4,784 (9.4)
	Yorkshire and Humber	64,614 (11.9)	6,208 (12.2)
Amoxicillin (%)	False	487,387 (89.7)	43,720 (85.6)
	True	56,204 (10.3)	7,353 (14.4)
Amoxicillin–clavulanic acid (%)	False	524,526 (96.5)	45,323 (88.7)
	True	19,065 (3.5)	5,750 (11.3)
Clindamycin (%)	False	542,279 (99.8)	49,773 (97.5)
	True	1,312 (0.2)	1,300 (2.5)
Cephalosporins (%)	False	533,259 (98.1)	48,402 (94.8)
	True	10,332 (1.9)	2,671 (5.2)
Fluoroquinolones (%)	False	536,127 (98.6)	49,059 (96.1)
	True	7,464 (1.4)	2014 (3.9)
PPI (%)	False	339,148 (62.4)	23022 (45.1)
	True	204,443 (37.6)	28,051 (54.9)

CIPS: continuous in-patient spell; IMD: index of multiple deprivation; n: number; OR: odds ratio; PPI: proton pump inhibitor; SD: standard deviation.

^a^ Comorbidities exclude cancer and renal disease.

We present total numbers and percentage breakdowns for categorical variables and means and standard deviation for continuous variables. Where a percentage is given the denominator is the total population (i.e. 543,591 for controls and 51,073 for cases).

**Figure 1:**
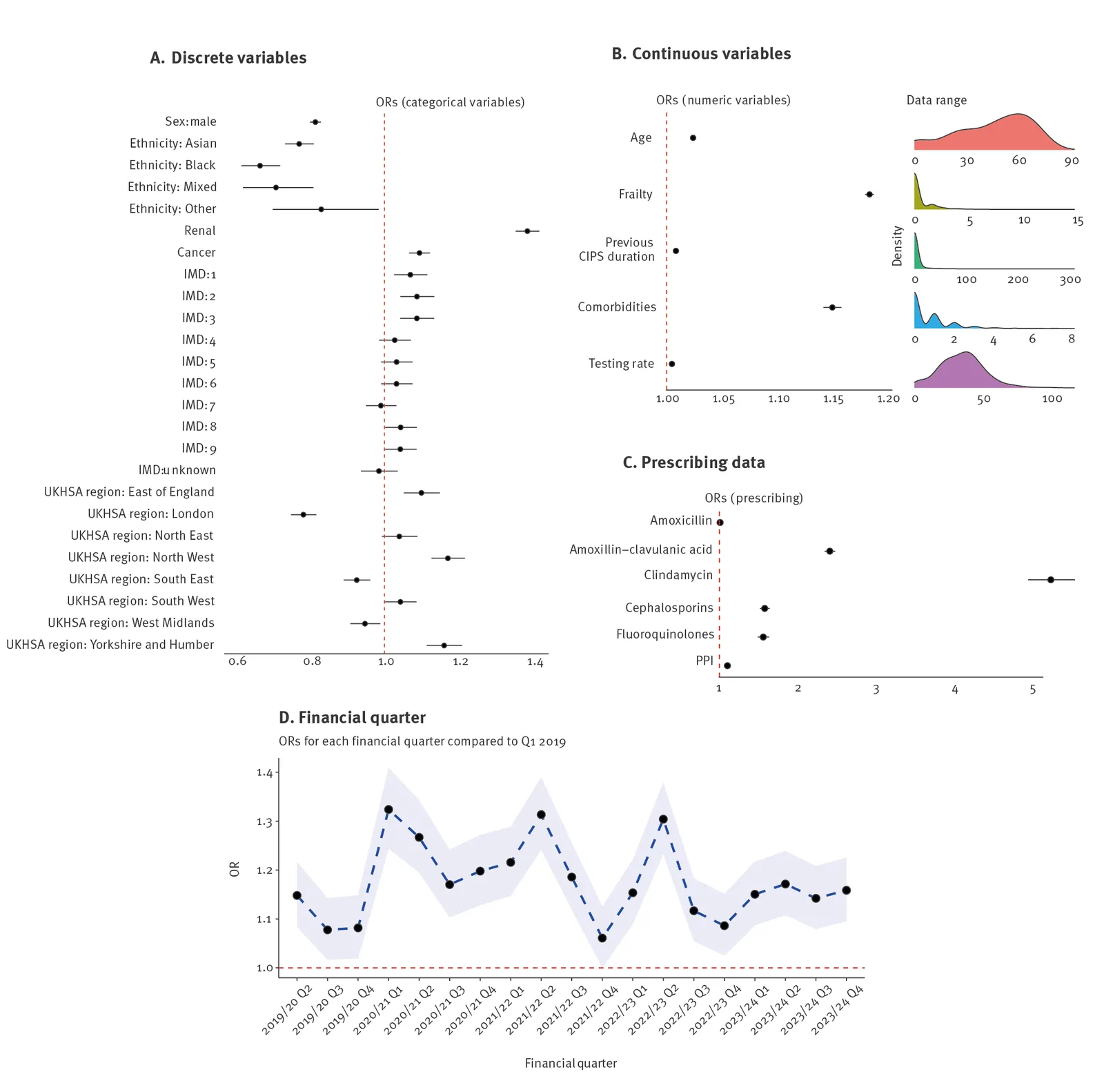
Odds ratios for CDI from the logistic regression model for (A) discrete variables (B) continuous variables (C) prescribing data and (D) financial quarters, England, 2019–2024 (n = 594,664 cases and controls)

CDI: *Clostridium difficile* infection; CIPS: continuous in-patient spell; FQ: financial quarter; IMD: index of multiple deprivation; OR: odds ratio; PPI: proton pump inhibitor; UKHSA: United Kingdom Health Security Agency.

Table 2 and Figure 1 describe the ORs of CDI from the full logistic regression model, stratified by CO and HO. The univariable analysis is also presented in Table 2. In the multivariable analysis, for all cases, almost all factors showed statistical significance (p < 0.001) except ‘other’ ethnicity, various IMD deciles (cf.d with IMD 10), various financial quarters (cf.d with Q1 2019), the North East, South West and West Midlands regions (cf.d with East Midlands) and amoxicillin use (Table 2).

**Table 2:** Odds ratios and p values of *Clostridium difficile* infection for each factor, adjusted for all other variables included in the model, as well as in the univariate models, England, 2019–2024 (n = 594,664 cases and controls)

Covariate			Multivariate analysis						CO vs HO cases	Univariate	
			All cases		CO cases		HO cases				
			OR	p value^a^	OR	p value^a^	OR	p value^a^	p value^a^	OR	p value^a^
Age^b^			1.03	< 0.001	1.03	< 0.001	1.03	< 0.001	0.178	1.04	< 0.001
Sex: male^c^			0.82	< 0.001	0.74	< 0.001	0.96	< 0.001	< 0.001	0.90	< 0.001
Ethnicity		Asian	0.77	< 0.001	0.74	< 0.001	0.83	< 0.001	0.020	0.41	< 0.001
		Black	0.67	< 0.001	0.57	< 0.001	0.79	< 0.001	< 0.001	0.36	< 0.001
		Mixed	0.71	< 0.001	0.64	< 0.001	0.83	0.067	0.045	0.27	< 0.001
		Other	0.83	0.032	0.78	0.034	0.91	0.464	0.398	0.34	< 0.001
		Unknown	1.58	< 0.001	1.61	< 0.001	1.53	< 0.001	0.352	1.03	0.335
Financial quarter		2019/20 Q2	1.15	< 0.001	1.2	< 0.001	1.08	0.083	0.102	1.17	< 0.001
		2019/20 Q3	1.08	0.012	1.04	0.356	1.14	0.003	0.101	1.1	< 0.001
		2019/20 Q4	1.08	0.010	1.05	0.252	1.14	0.006	0.175	1.08	0.011
		2020/21 Q1	1.32	< 0.001	1.44	< 0.001	1.17	0.002	0.001	1.57	< 0.001
		2020/21 Q2	1.27	< 0.001	1.35	< 0.001	1.16	0.002	0.011	1.41	< 0.001
		2020/21 Q3	1.17	< 0.001	1.21	< 0.001	1.12	0.015	0.207	1.25	< 0.001
		2020/21 Q4	1.2	< 0.001	1.18	< 0.001	1.23	< 0.001	0.532	1.28	< 0.001
		2021/22 Q1	1.22	< 0.001	1.24	< 0.001	1.18	< 0.001	0.439	1.25	< 0.001
		2021/22 Q2	1.31	< 0.001	1.36	< 0.001	1.24	< 0.001	0.101	1.4	< 0.001
		2021/22 Q3	1.19	< 0.001	1.16	< 0.001	1.23	< 0.001	0.268	1.28	< 0.001
		2021/22 Q4	1.06	0.049	0.99	0.828	1.16	0.001	0.009	1.16	< 0.001
		2022/23 Q1	1.15	< 0.001	1.1	0.018	1.23	< 0.001	0.045	1.31	< 0.001
		2022/23 Q2	1.3	< 0.001	1.29	< 0.001	1.33	< 0.001	0.614	1.5	< 0.001
		2022/23 Q3	1.12	< 0.001	1.02	0.683	1.26	< 0.001	< 0.001	1.23	< 0.001
		2022/23 Q4	1.09	0.005	0.94	0.088	1.31	< 0.001	< 0.001	1.19	< 0.001
		2023/24 Q1	1.15	< 0.001	1.07	0.063	1.27	< 0.001	0.005	1.3	< 0.001
		2023/24 Q2	1.17	< 0.001	1.15	< 0.001	1.2	< 0.001	0.445	1.36	< 0.001
		2023/24 Q3	1.14	< 0.001	1.12	0.003	1.18	< 0.001	0.349	1.28	< 0.001
		2023/24 Q4	1.16	< 0.001	1.22	< 0.001	1.08	0.111	0.034	1.28	< 0.001
Frailty			1.2	< 0.001	1.12	< 0.001	1.28	< 0.001	< 0.001	1.4	< 0.001
Renal disease			1.38	< 0.001	1.17	< 0.001	1.7	< 0.001	< 0.001	2.54	< 0.001
Cancer			1.09	< 0.001	0.93	< 0.001	1.36	< 0.001	< 0.001	1.25	< 0.001
Comorbidities^d^			1.17	< 0.001	1.09	< 0.001	1.27	< 0.001	< 0.001	1.7	< 0.001
Testing rate^e^			1.01	< 0.001	1.01	< 0.001	1	< 0.001	0.019	1.01	< 0.001
IMD	1		1.07	0.002	0.98	0.358	1.23	< 0.001	< 0.001	1.02	0.272
	2		1.09	< 0.001	1.03	0.312	1.19	< 0.001	< 0.001	0.95	0.025
	3		1.09	< 0.001	1.01	0.821	1.22	< 0.001	< 0.001	0.97	0.163
	4		1.03	0.201	0.98	0.433	1.11	0.002	0.004	0.998	0.937
	5		1.03	0.128	0.97	0.311	1.13	< 0.001	< 0.001	1.02	0.341
	6		1.03	0.13	0.97	0.219	1.15	< 0.001	< 0.001	1.03	0.193
	7		0.99	0.634	0.94	0.026	1.08	0.027	0.002	1.01	0.628
	8		1.04	0.042	1	0.934	1.11	0.001	0.013	1.06	0.006
	9		1.04	0.047	1.03	0.204	1.06	0.087	0.577	1.04	0.038
	Unknown		0.99	0.534	0.94	0.0642	1.07	0.109	0.016	0.73	< 0.001
Region	East of England		1.1	< 0.001	1.01	0.79	1.26	< 0.001	< 0.001	0.99	0.731
	London		0.78	< 0.001	0.73	< 0.001	0.86	< 0.001	< 0.001	0.63	< 0.001
	North East		1.04	0.0952	0.98	0.404	1.14	< 0.001	< 0.001	1.02	0.438
	North West		1.17	< 0.001	1.16	< 0.001	1.18	< 0.001	0.729	1.3	< 0.001
	South East		0.93	< 0.001	0.95	0.056	0.89	< 0.001	0.081	0.97	0.11
	South West		1.04	0.045	1.07	0.013	1	0.95	0.127	1.06	0.004
	West Midlands		0.95	0.013	0.93	0.010	0.98	0.591	0.206	0.89	< 0.001
	Yorkshire and Humber		1.16	< 0.001	1.08	0.003	1.28	< 0.001	< 0.001	0.999	0.976
Antibiotics	Amoxicillin		1.01	0.482	1.1	< 0.001	0.90	< 0.001	< 0.001	1.46	< 0.001
	Amoxicillin–clavulanic acid		2.41	< 0.001	2.98	< 0.001	1.66	< 0.001	< 0.001	3.49	< 0.001
	Clindamycin		5.22	< 0.001	7.11	< 0.001	2.7	< 0.001	< 0.001	10.8	< 0.001
	Cephalosporins		1.58	< 0.001	1.76	< 0.001	1.34	< 0.001	< 0.001	2.85	< 0.001
	Fluoroquinolones		1.56	< 0.001	1.75	< 0.001	1.24	< 0.001	< 0.001	2.95	< 0.001
PPI			1.1	< 0.001	1.2	< 0.001	0.98	0.233	< 0.001	2.02	< 0.001
Total CIPS duration			1.01	< 0.001	1.01	< 0.001	1.01	< 0.001	0.28	1.01	< 0.001
Renal disease total CIPS duration			0.998	< 0.001	0.998	< 0.001	0.997	< 0.001	< 0.001	NA	NA
Cancer total CIPS duration			0.998	< 0.001	0.999	< 0.001	0.997	< 0.001	< 0.001	NA	NA

CIPS: continuous in-patient spell; CO: community onset; HO: hospital onset; IMD: index of multiple deprivation; OR: odds ratio; PPI: proton pump inhibitor.

^a^ Results were considered statistically significant if p < 0.001.

^b^ Per additional year.

^c^ Sex was included as a binary variable as further described in Supplement 2 and there were no data missing on sex in the table.

^d^ Comorbidities exclude cancer and renal disease.

^e^ Per additional stool sample tested per 1,000 admissions.

Results show ORs and p values for models with all cases, CO cases and HO cases considered, as well as univariate ORs for all cases. We also show the p-value comparing CO and HO values. Reference populations for sex were female; ethnicity, ‘White’; IMD, IMD 10 (least deprived); financial quarter, 2019/20 Q1; region, East Midlands.

Reference populations for sex were female; ethnicity, ‘White’; IMD, IMD 10 (least deprived); FQ, 2019/20 Q1; region, East Midlands.

Positive associations were seen for age (OR: 1.03 per additional year of age), and all comorbidities included, especially renal disease (OR: 1.38). Non-white ethnicities (cf.d with White) and male sex (cf.d with female) all had negative associations with CDI.

For aggregated variables, the effect was weaker. Testing rates had an OR of 1.01 per additional stool sample tested per 1,000 admissions. As deprivation increased, the OR slightly increased. The regions with the highest ORs (reference East Midlands) were the North West (OR: 1.17) and Yorkshire and Humber (OR: 1.16). The regions with the lowest ORs were London (OR: 0.78) and the South East (OR: 0.93).

All antibiotics modelled except for amoxicillin showed a strong positive association with the odds of CDI. Fluoroquinolones, cephalosporins and amoxicillin–clavulanic acid had ORs of 1.56, 1.58 and 2.41 respectively. Clindamycin showed the largest association with an OR of 5.22.

The ORs for financial quarters (Figure 1D) showed all quarters having higher odds than 2019/20 Q1, varying from 1.06 to 1.32. We did not however see a clear increasing linear trend.

There were some significant differences in CO and HO cases. For antibiotics, the OR was always substantially higher for CO than HO. For example, CO clindamycin had an OR of 7.11 compared with 2.70 for HO. For all considered comorbidities, the ORs were higher for HO than for CO. For example, the OR for cancer was 0.93 (negative association) for CO but 1.36 (positive association) for HO.

### Fitting 2019 and predicting forwards

**Figure 2:**
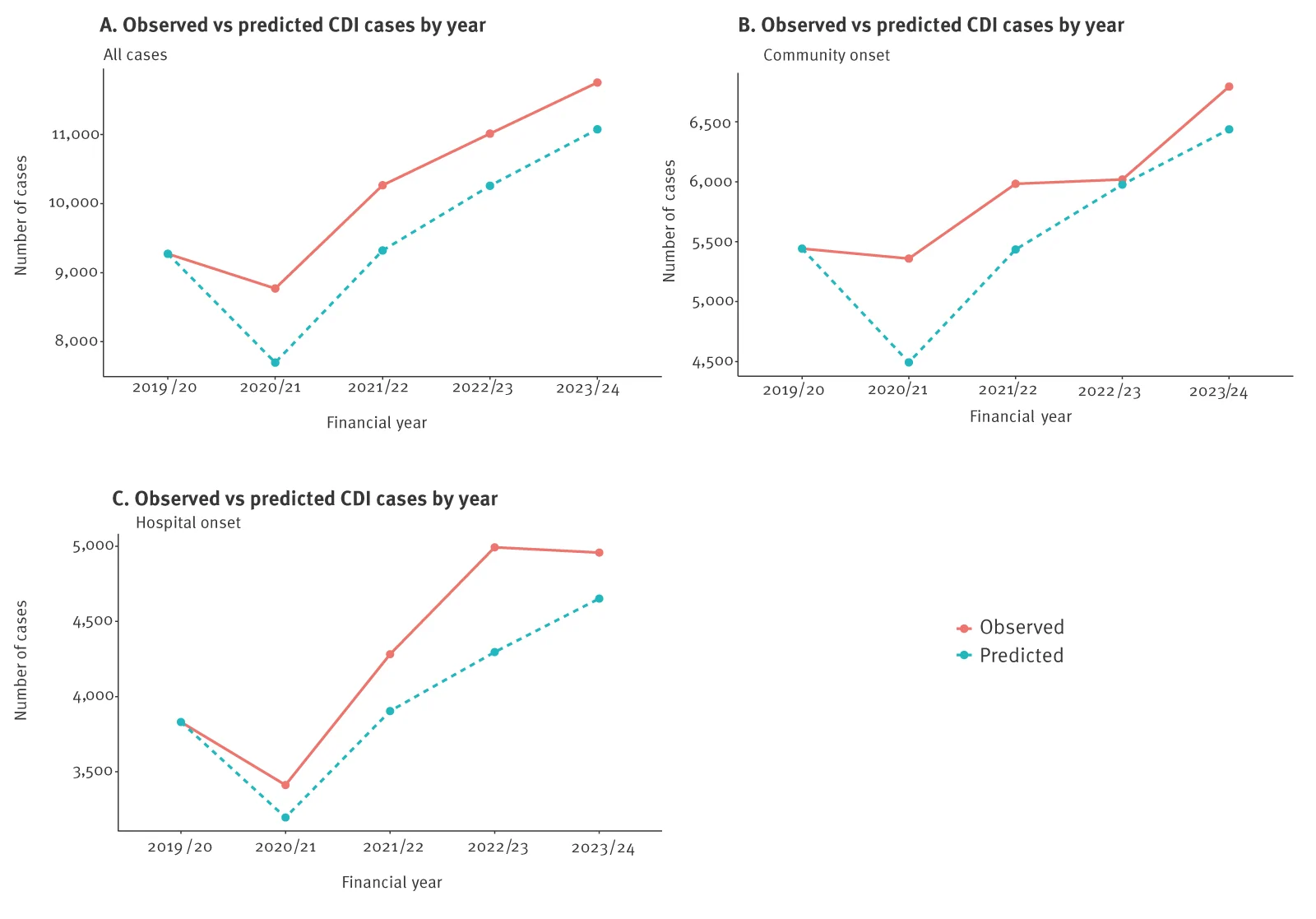
Predicted and observed cases, including (A) all cases, (B) cases with community onset and (C) cases with hospital onset, using the full logistic regression model trained on 2019/20 data and predicting 2020/21 onwards, England, 2019–2024

The full model predicts an overall increase in CDI cases based on underlying changes to the characteristics of the hospitalised population, but it does not fully explain the variation in observed case numbers (Figure 2). Total predicted cases rose from 9,273 in 2019/20 to 11,074 in 2023/24. However, observed cases rose more steeply to 11,752 cases. Therefore 94% of the cases are explained using this model, as described in Supplement 4. There is a significant step change around 2020/21 where the model underpredicts cases (88% explained, Supplement 4) and that underprediction continues for future years.

CDI: *Clostridium difficile* infection.

Table E1 of Supplement 4 gives the numbers corresponding to this figure.

This trend is also true for both CO and HO cases, with 94.7% and 93.8% of cases explained respectively. The HO model fits better over the COVID pandemic period than the CO, with 94% explained for HO compared with 84% for CO. The HO model fits less well in 2022/23, explaining only 86% of cases compared with 99% explained by the CO model.

**Figure 3:**
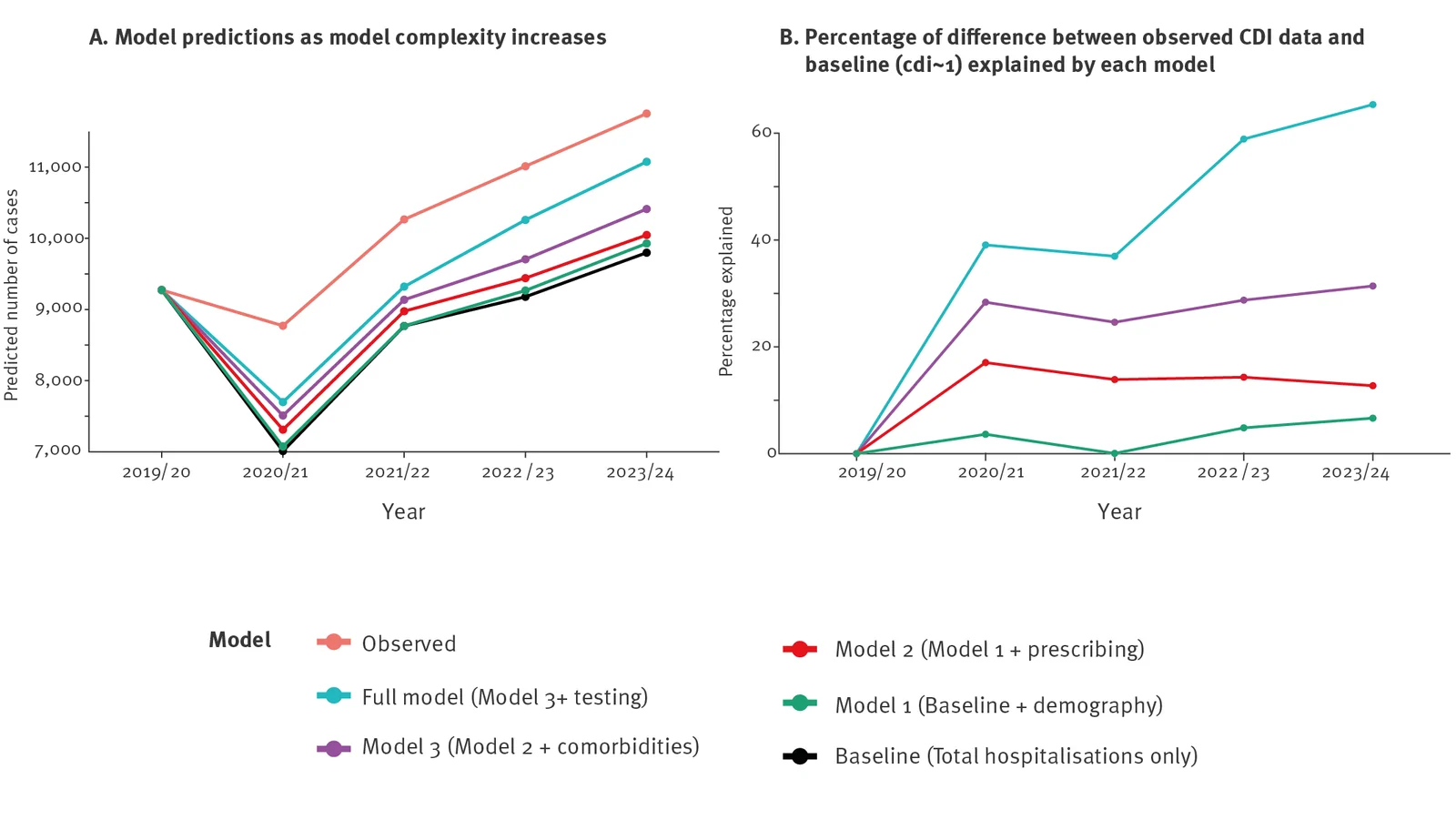
(A) breaking the full model into sub-models, down to the baseline model, all fitted to 2019/20 data and predicting CDI case numbers from 2020/21 onwards, with findings compared to observed data and (B) share of the percentage difference between the baseline model output and observed data, explained by each (sub-)model, England, 2019–2024

Figure 3 shows testing and comorbidities as the key drivers of the rise in the model. Corresponding data points are in Supplement 4. The baseline model gives the overall shape of our prediction and shows a rise in cases since a low in 2020/21. In 2023/24 there were 9,797 baseline cases compared with 11,752 observed. Adding demography (Model 1) predicts 9,923 cases in 2023/24, explaining 6.4% of the difference between baseline and observed. Adding prescribing (Model 2) increases predicted cases in 2023/24 to 10,043, explaining 12.6%. Adding comorbidities (Model 3) increases the predicted number of cases to 10,410. This is 31.3% of the difference between baseline and observed. Adding testing rates into the model further increases the prediction to 11,074 cases in 2023/34, explaining 65.3% of the difference between baseline and observed.

CDI: *Clostridium difficile* infection.

Table E2 of Supplement 4 gives the numbers corresponding to this figure.

## Discussion

The ORs that we see in the model are in line with those observed in previous studies [6]. Increased age, frailty and comorbidities are all known risk factors for CDI, the scale of which is seen in the ORs. For example, odds of CDI increase 3% per year of age and 1% per night spent in hospital. We also see that there are lower odds of infection for males and non-white ethnicities. Regional differences also tie in with previous work [9] and we encourage further research into unmodelled drivers of regional difference such as demographic factors, individual-level deprivation or local hospital conditions.

Primary care antibiotic consumption provided the highest discrete risk factor for the individual, especially clindamycin (OR of 5.22) confirming known associations with this antibiotic [20]. All other antibiotics except amoxicillin increased the risk of CDI significantly. Therefore, extra care must be taken to protect individuals who have had previous exposure to any of these antibiotics, aligning with current research emphasising the impact of antibiotics on the gut microbiome [21].

The difference between the ORs for CO and HO must be explained by context as well as epidemiology. For example, a cancer patient is likely to be in hospital regularly and therefore, is more likely to pick up a CDI in the hospital rather than the community vs someone with average hospital exposure. Conversely, antibiotics usage data are only for community prescribing and exclude antibiotic exposure information for anyone who took antibiotics in hospital or was prescribed via outpatient services (see limitations). Moreover, we do not, in this paper, distinguish CO cases into hospital associated and community associated cases.

Temporal analysis modelling showed that changes in the frequency of risk factors known to be associated with CDI risk would predict an increase in CDI cases, but not to the extent that we observed, and sources of variation exist which were not captured in this model. It does not support changing demography, increased ageing and increased antibiotic use as substantial drivers of the modelled increase. The case increase that is explained by the model is predicted by increases in overall hospitalisation rates, comorbidities − especially renal disease, increased frailty, and testing rate increases.

The model significantly underpredicted cases in 2020/21 and this carried forward to later years. Although causation is not clear, there might be a lasting effect of the COVID-19 pandemic that is not captured by our model, but which is ongoing. There have been significant changes in hospital pressures causing treatment delays and an overstretched workforce potentially leading to lapses in infection prevention and control (IPC), and increased transmission. Moreover, during the pandemic, changes in hospital antibiotic prescribing may have occurred that are not captured here (see limitations). These are counterbalanced by reductions in social mixing, an increased public awareness of hand hygiene and reduced hospitalisations. This has led studies in the UK and internationally to find mixed impacts of the pandemic on case numbers [22-27]. Moreover, international CDI case rates do not follow the same increases seen in the UK [14,15], implying that this is not a universal impact of COVID-19. Further research is therefore needed to understand the dynamics that caused these results and determine the missing data that will explain the difference between the observed and predicted cases.

The comparison population for this study are all hospitalised patients. This has two limitations. Firstly, there may be hospitalised patients whose risk of CDI is unrelated to their hospitalisation but might impact the ORs. Secondly, the hospitalised population will have changed significantly over time. If, for example, control cases were reduced, then the comparative chance of contracting CDI in the logistic regression would increase.

This model shows correlation, not causation and care must be taken when interpreting the results. In particular, the association between testing rates and risk of CDI should be interpreted with care. Although an increase in testing might explain a rise in cases, it may also be a response to a rise in cases. It is worth noting that although testing rates have increased at a national level, this may not be the case in all trusts with admitted patients who were included in the study. At a national level, the test positivity rate is relatively unchanged over the past 5 years and shows consistent seasonal variation, with peaks in Q3 (October–December) and troughs in Q1 (April–June) [9]. Because the positivity rates have not declined as testing rates have increased, the association between testing rates and risk of CDI observed in this study might indicate that testing has increased in response to an increase in the underlying risk of CDI among people presenting with diarrhoea, and clinicians’ testing thresholds have remained constant. The increase in both testing and CDI rates might be also caused by an increase in overall diarrhoea causing illnesses that may also explain why both renal disease and frailty (which includes incontinence) have increased over time.

In this model we do not consider recurrent infections; each infection or hospitalisation is considered as a separate data point. This was the most parsimonious assumption we could make. Moreover, we do not look at second order effects such as the impact of antibiotics broken down by age. Although the end of the 2019/20 financial year coincided with the initial emergence of the pandemic, any resulting impact on the baseline numbers of infections is likely minimal. Lastly, we do not have the ribotyping of our cases. In England, approximately half of *C. difficile* reported cases were submitted to the CDRN for ribotyping [13]. However, the UKHSA has concluded that clonal expansion is not the likely cause of the increase [9,12].

Antibiotic data have two key limitations. Firstly, we were unable to access any individual data on hospital prescriptions; meaning we may be missing key causal links. Our analysis showed that primary care antibiotics had very large ORs and there is no reason to believe the impact would not be similar for secondary care. Moreover, as the majority of our cases were HO or hospital associated, any antibiotics prescribed in hospital that were the direct cause of the *C. difficile* infection could not be included. For example, our CDI cases have significantly longer hospital exposure (26.7 previous nights in hospital in the preceding 12 months, compared with 4.2 for controls) which increases their risks of consuming antibiotics and being exposed to this risk. If secondary care antibiotics were included this might reduce the ORs for other factors, such as age, which may be associated with antibiotic prescribing. Lastly, we are unable to see the extent to which changes in secondary care prescribing at the individual level have changed over time and to what extent this might explain the rise in CDI cases. Although aggregated data are available, a hospital which prescribes more antibiotics per person is a vastly different risk factor to an individual’s antibiotic exposure and is therefore not suitable for this work.

Secondly, the date of claiming a prescription for primary care data means it is possible that a prescription occurs after the CDI incident or hospitalisation episode. However, to exclude this possibility we would lose cases where antibiotic prescribing occurs close to infection which would underestimate the effect of prescribing on the odds of infection. These issues underscore the need for individual level prescribing data in primary and secondary care with dates that reflect exposure to improve the accuracy of our modelling.

Deprivation as a risk factor is not captured at the individual level, nor does it vary over time, this study may therefore underestimate its impact. Hospital level risk factors such as care in unconventional places (‘corridor care’), staff pressures, or IPC compliance do not have sufficient data to include in the model, and they may be explanatory factors. We exclude any individuals without a hospitalisation episode (less than 1.9%, Supplement 2). We include the small number of CDI cases that have a HES match which is significantly further away from their CDI episode. For example, 9% of cases had their CDI episode more than 84 days before or after their hospitalisation, as described in Supplements 2 and 5. This may mean that their comorbidities and regional data may have changed in this time. However, this allows us to include as much information on true community cases as we can.

## Conclusion

The study shows that a substantial proportion of the increase of cases of CDI could be explained by changes in numbers of hospitalisations, comorbidities and testing rates. Conversely, the model allows us to say that an ageing population and recent antibiotic use in primary care explain a smaller proportion of the rises we see. There is a significant underestimation of the number of cases predicted, beginning in the COVID-19 pandemic (2020/21) and continuing onwards. We hope that further work can explore what might be underpinning this unexplained rise in cases, and that further data on hospital pressures and hospital antibiotic prescribing might soon be available to improve the capabilities of this model and better understand the key drivers associated with the recent increase in CDI incidence.

## Data Availability

All data were collected within statutory approvals granted to United Kingdom Health Security Agency for infectious disease surveillance and control. Information was held securely and in accordance with the Data Protection Act 2018 and Caldicott guidelines. The data that support the findings of this study are available from NHS Digital, but restrictions apply to the availability of these data, which were used under license for the current study, and so are not publicly available. Data are however available from the authors upon reasonable request and with permission of NHS Digital. Code to run all logistic regression models, including the prediction models and plotting code is available at https://github.com/ukhsa-collaboration/CDI_LR_external although the data to run this model is sensitive and therefore not publicly available.

## Supplementary Data

127000 Supplement 1

## Supplementary Data

90000 Supplement 2

## Supplementary Data

150000 Supplement 3

## Supplementary Data

88000 Supplement 4

## Supplementary Data

127000 Supplement 5
