# Supplementary material for: Factors driving increases in Clostridioides difficile infection rates in England: a national case−control study, 2019 to 2024
Source: Euro Surveill. 2026 Sep 17;31(37):2500888. doi: 10.2807/1560-7917.ES.2026.31.37.2500888 (PMC13589211; doi:10.2807/1560-7917.ES.2026.31.37.2500888)
Supplement: Supplementary file 1 [file 25-00888_WHITELEY_Supplement1.pdf]

This supplementary material is hosted by Eurosurveillance as supporting information alongside the article 'Factors driving increases in Clostridioides difficile infection rates in England: A national case-control study, 2019 to 2024', on behalf of the authors, who remain responsible for the accuracy and appropriateness of the content. The same standards for ethics, copyright, attributions and permissions as for the article apply. Supplements are not edited by Eurosurveillance and the journal is not responsible for the maintenance of any links or email addresses provided therein.

Appendix D: Comparing a 1% sample of controls to a 0.1% sample

We want to test the sensitivity of the size of the control cases we used. We currently use a 1% sample and use the input weights=100 for non-cdi cases. To test this hypothesis, we created a smaller dataset by taking a 10% sample of our control cases, leaving 0.1% of all non-cdi hospitalisations. We then use weights=100 in the *glm* function and calculate odds ratios. The comparison table is below.

Most variables do not show any statistically significant change. There are a few statistically significant differences for some financial quarters and notably clindamycin and cephalosporin use.

	OR 1% sample	OR 0.1% sample	p_value (ORs are different)
(Intercept)	0.0001	0.0001	0.0280
Age	1.0264	1.0260	0.2453
Sex: Male	0.8150	0.8281	0.2139
Ethnicity: Asian	0.7716	0.7072	0.0147
Ethnicity: Black	0.6670	0.6454	0.5584
Ethnicity: Mixed	0.7095	0.5868	0.0469
Ethnicity: Other	0.8306	0.8388	0.9364
Ethnicity: Unknown	1.5761	1.4849	0.1114
Financial Quarter: 2019/20 Q2	1.1483	1.0044	0.0013
Financial Quarter: 2019/20 Q3	1.0780	0.9755	0.0179
Financial Quarter: 2019/20 Q4	1.0819	0.9913	0.0428
Financial Quarter: 2020/21 Q1	1.3237	1.2826	0.4804
Financial Quarter: 2020/21 Q2	1.2667	1.1627	0.0426
Financial Quarter: 2020/21 Q3	1.1706	1.0424	0.0067
Financial Quarter: 2020/21 Q4	1.1981	1.1472	0.3174
Financial Quarter: 2021/22 Q1	1.2159	1.0574	0.0008
Financial Quarter: 2021/22 Q2	1.3137	1.1687	0.0041
Financial Quarter: 2021/22 Q3	1.1860	1.0614	0.0075
Financial Quarter: 2021/22 Q4	1.0611	0.9378	0.0037
Financial Quarter: 2022/23 Q1	1.1537	1.0398	0.0118
Financial Quarter: 2022/23 Q2	1.3044	1.2760	0.5832

Financial Quarter: 2022/23 Q3	1.1170	0.9211	0.0000
Financial Quarter: 2022/23 Q4	1.0866	0.9838	0.0176
Financial Quarter: 2023/24 Q1	1.1507	1.0570	0.0384
Financial Quarter: 2023/24 Q2	1.1717	1.0658	0.0192
Financial Quarter: 2023/24 Q3	1.1425	1.0914	0.2610
Financial Quarter: 2023/24 Q4	1.1590	1.1138	0.3264
Frailty	1.2027	1.1959	0.0343
Renal Disease	1.3826	1.3513	0.1667
Total cips duration	1.0093	1.0091	0.0770
Cancer	1.0938	1.0476	0.0198
Comorbidities	1.1656	1.1904	0.0002
Testing rate	1.0054	1.0049	0.2421
IMD: 1	1.0690	1.1612	0.0059
IMD: 2	1.0867	1.1401	0.1124
IMD: 3	1.0865	1.0786	0.8102
IMD: 4	1.0273	1.0556	0.3636
IMD: 5	1.0323	1.1061	0.0195
IMD: 6	1.0321	1.1130	0.0106
IMD: 7	0.9901	1.0528	0.0384
IMD: 8	1.0432	1.0972	0.0870
IMD: 9	1.0423	1.0307	0.7042
IMD: Unknown	0.9843	0.9432	0.2387
Region: East of England	1.0985	1.1419	0.2223
Region: London	0.7828	0.7313	0.0301
Region: North East	1.0396	0.9976	0.2103
Region: North West	1.1695	1.1996	0.3572
Region: South East	0.9256	0.9165	0.7198
Region: South West	1.0424	1.1178	0.0174
Region: West Midlands	0.9478	0.9273	0.4749
Region: Yorkshire and Humber	1.1596	1.0772	0.0123
Amoxicillin	1.0091	0.9688	0.0261
Co-Amoxiclav	2.4064	2.5119	0.0360
Clindamycin	5.2225	3.9630	0.0000
Cephalosporins	1.5774	1.7430	0.0005
Fluoriquinolones	1.5587	1.5680	0.8565
PPI	1.1025	1.0731	0.0417
Renal Disease * Total cips duration	0.9977	0.9976	0.5545
Cancer * Total cips duration	0.9979	1.0003	0.0000

Classified as ECDC NORMAL
