# Supplementary material for: Factors driving increases in Clostridioides difficile infection rates in England: a national case−control study, 2019 to 2024
Source: Euro Surveill. 2026 Sep 17;31(37):2500888. doi: 10.2807/1560-7917.ES.2026.31.37.2500888 (PMC13589211; doi:10.2807/1560-7917.ES.2026.31.37.2500888)
Supplement: Supplementary file 2 [file 25-00888_WHITELEY_Supplement2.pdf]

This supplementary material is hosted by Eurosurveillance as supporting information alongside the article 'Factors driving increases in Clostridioides difficile infection rates in England: A national case-control study, 2019 to 2024', on behalf of the authors, who remain responsible for the accuracy and appropriateness of the content. The same standards for ethics, copyright, attributions and permissions as for the article apply. Supplements are not edited by Eurosurveillance and the journal is not responsible for the maintenance of any links or email addresses provided therein.

Appendix A: Data sources, missingness and exclusions

Data sources

We used data from the following sources.

Case data: The test methods used to confirm a *C. difficile* infection vary between NHS Acute Trusts, typically following a two- or three-step algorithm that confirms the presence of the *C. difficile* glutamate dehydrogenase (GDH) antigen and/or a toxigenic strain of *C. difficile,* as well as the presence of *C. difficile* toxins in a faecal specimen, as recommended in UKHSA Standards for Microbiological Investigations [1]. Children under 2 are excluded from this dataset. Only symptomatic patients are tested, i.e. those with diarrhoea, and include patients in both hospital and community healthcare settings (e.g. a General Practitioner's (GP) surgery). Details on the quality and methodology of the data once collected can be seen in [2].

HES: We joined reported CDI cases to their hospital admission using patient NHS number, first name and surname. We considered continuous in-patient spells (CIPs) for each admission. Where a CDI case had multiple admissions to hospital, we chose the spell that is closest to their CDI diagnosis which may have been before or after. HO cases were those that had positive samples taken two days or more into their hospital spell where admission is counted as day 0, but before discharge [3]. Everything else was CO [3]. We recorded the closeness of each CDI episode to a hospitalisation episode. 67% of our CDI cases occur within the period of their hospitalisation (not necessary defined as HO). 84% of CDI cases have their closest hospitalisation episode within 30 days of their CDI diagnosis (either before or after), 91% are within 84 days and 96% are within 1 year. Sensitivity analysis was conducted excluding positive CDI results >84 days before or after a hospitalisation spell (Appendix B) and the results were broadly similar. To maximise the data availability, we included these cases.

Frailty and comorbidities: These were estimated from the ICD-10 codes listed against the hospital spell. Frailty was calculated as the Dr Foster Fragility metric from Soong et al [4] for which there are seven groups of ICD-10 codes as follows: cognition, mobility, falls, malnutrition, incontinence, dependencies and mood. For comorbidities, we derived a modified Charlson Comorbidity index [5] by removing age, cancer and renal components. Age was considered separately. Cancer (lymphoma, leukaemia and solid tumour) and renal disease (moderate to severe chronic kidney disease) were deemed epidemiologically important and therefore picked out separately from this index. We then summed the remaining factors in the index without previous weighting to give a measure of other comorbidities. We adapted the python package *comorbidipy* [6] to extract the ICD-10 codes and implement the metric.

Previous hospitalisation: For each previous hospitalisation spell in the preceding 12 months to the matched HES record, we summed the number of overnight bed-days in hospital (Total CIPS duration). We found that cancer and renal disease were sensitive to the inclusion of previous hospitalisation, and we therefore consider the second order interaction of duration and cancer and renal disease.

Testing: Trust-level reports of total stool samples tested for CDI were taken from quarterly mandatory laboratory returns data (QMLR), submitted as part of the mandatory surveillance system for CDI. This data was normalised by the number of admissions to the hospital [7] giving a metric of tests per 1000 admissions.

Prescribing: Individual level primary care antibiotics and Proton pump inhibitor (PPI) prescribing data comes from the 'Medicines Dispensed in Primary Care' (MDPC) dataset [8]. Prescribing that was chosen were: antibiotics deemed high risk of CDI (Co-Amoxiclav, Clindamycin, Cephalosporins and Fluoroquinolones), Amoxicillin (as a reference antibiotic), and PPIs. This data gives the month that the prescription was submitted to NHS Business Services Authority for processing. For each antibiotic class, we calculated the difference between the month of prescription and the month of CDI onset (for cases) or hospital admission (for controls). If this difference was 6 months or less, then this antibiotic variable is set to True. Details of the BNF codes used as well as sensitivity analysis on the choice of six months can be seen in appendix C. Individual level secondary care data were not available for this study (see limitations).

Deprivation: We used the 2019 index of multiple deprivation (IMD)[9] which is the deprivation score of the lower super output area (LSOA) of the postcode registered for the patient. This gives a score is from 1-10 where IMD 10 is the least deprived. The IMD is treated as a factor with the default IMD level as 10. Missing, unmatched, or non-England postcodes were recorded in a category 'missing'. This metric is static over time.

Sex data: Sex was recorded as 'Male' or 'Female', with 'Female' as the reference group. Cases where sex was recorded as something other than these two categories appear to have been mostly due to poor data recording. Because the small number of such cases was found to distort our prediction models, these cases were excluded.

Age & Ethnicity data: We removed any cases reported for individuals older than 110 years, as this likely indicated an error in the data. We consolidated ethnicity into the following groups: 'White', 'Black', 'Asian', 'Mixed', 'Other/Unknown'. The reference group for ethnicity was White. Ethnicity data was enhanced using the CHIME methodology as detailed in the UKHSA quality and methodology information for health care acquired infection reports [2].

Region: The UKHSA region of the trust. The default region is East Midlands. This is first alphabetically and incidence are mid-range nationally.

Financial year and quarter: For cases, this is the date of their first positive CDI result. For controls, the date of their admission. This is a factor in the model i.e. each time point is independent. In HES, the financial year is 1st April to 31st March.

Data missingness:

There were 71052 initial CDI records within the study period. Of these, 1250 (1.76%) are unable to be joined with any hospitalisation episode within HES and therefore cannot be used. These might not merge due to errors in NHS numbers or names; or these are individuals who never went to hospital for their CDI episode, nor went to hospital for any other reason in the study period. Moreover 289 CDI incidents joined with a HES record but there were issues with the date recorded in HES and were discarded.

Having merged with HES, and selected the closest spell, we have 69513 cases and 815327 controls from the financial years 2019/20 up to 23/24 giving a total of 884840 data points.

We exclude any non-acute trusts as defined in the UKHSA fingertips dataset [7].

We exclude trusts “R0D” and “RJ1” as there is significant missing sex data which is missing more in the controls than the cases.

We exclude 36 trusts with missing reports of numbers of stool samples tested for CDI as missingness in the testing data increases over time. This excludes trusts “R0A”, "ROB", "RA7", "RAE", "RAJ", "RAS", "RBT", "RD8", "RDE", "RDU", "REN", "REP", "RF5", "RGP", "RGR", "RH5", "RJN", "RJZ", "RLT", "RMP", "RN7", "RNN", "RNS", "RP4", "RPA", "RPY", "RQ3", "RQM", "RQW", "RRK", "RRV", "RTK", "RWD", "RWY", "RXK" & "RXR". This leaves 51294 cases and 544590 controls (595884 data points).

We remove any case with a previous stay of greater than 1 year. We remove anyone aged over 110 or where age is not recorded. We also removed the 19 cases and 161 controls where sex is not recorded as being male or female. The removed cases appear to be where data is poorly recorded, and the small numbers were found to distort our prediction models.

This leaves 51073 cases and 543591 controls (594664 data points). Overall, we excluded 28.1% of cases (19,979/71,052) and 33.3% (271,736/815,327) of controls.

Any missingness in categorical data, such as sex, ethnicity and IMD is included within a category ‘other’. Missingness in ICD-10 codes and antibiotic data is unknown but will be recorded as someone not having the condition/ not taken the antibiotic.

References

1. UK Health Security Agency. UK Standards for Microbiology Investigations [Internet]. 2025 [cited 2026 Mar 16]. Investigation of faecal specimens for Clostridioides difficile. Available from: https://www.rcpath.org/static/a98af0b3-c83c-47ad-83bf3615590b61da/UK-SMI-B-10i2-Investigation-of-faecal-specimens-for-Clostridioides-difficile-March-2025.pdf

2. GOV.UK [Internet]. [cited 2025 Oct 17]. Quality and methodology information (QMI) for healthcare-associated infections (HCAI) reports. Available from: https://www.gov.uk/government/statistics/hcai-qmi-report/quality-and-methodology-information-qmi-for-healthcare-associated-infections-hcai-reports

3. UK Health Security Agency. GOV.UK [Internet]. [cited 2025 Nov 12]. Mandatory enhanced MRSA, MSSA and Gram-negative bacteraemia, and Clostridioides difficile infection surveillance - Protocol version 4.4. Available from: https://hcaidcs.ukhsa.gov.uk/ContentManagement/LinksAndAnnouncements/HCAIDCS_Mandatory_Surveillance_Protocol_v4.4.pdf

Classified as ECDC NORMAL

4. Soong JTY, Gammall J, Liew D, Peden CJ, Bottle A, Bell D, et al. Dr Foster global frailty score: an international retrospective observational study developing and validating a risk prediction model for hospitalised older persons from administrative data sets. BMJ Open. 2019 Jun 1;9(6):e026759. doi:10.1136/bmjopen-2018-026759

5. Quan H, Sundararajan V, Halfon P, Fong A, Burnand B, Luthi JC, et al. Coding Algorithms for Defining Comorbidities in ICD-9-CM and ICD-10 Administrative Data. Medical Care. 2005 Nov;43(11):1130. doi:10.1097/01.mlr.0000182534.19832.83

6. vvcb. comorbidipy: Python package to calculate comorbidity scores and other clinical risk scores. [Python] [Internet]. [cited 2025 Oct 23]. Available from: https://github.com/vvcb/comorbidipy

7. Fingertips | Department of Health and Social Care [Internet]. [cited 2025 Oct 24]. Available from: https://fingertips.phe.org.uk/

8. NHS England. Medicines dispensed in Primary Care NHS Business Services Authority data product [Internet]. [cited 2025 Oct 17]. Medicines dispensed in Primary Care NHS Business Services Authority data product. Available from: https://digital.nhs.uk/data-and-information/data-tools-and-services/data-services/medicines-dispensed-in-primary-care-nhsbsa-data

9. GOV.UK [Internet]. [cited 2025 Nov 18]. English indices of deprivation 2019. Available from: https://www.gov.uk/government/statistics/english-indices-of-deprivation-2019
