# Supplementary material for: Factors driving increases in Clostridioides difficile infection rates in England: a national case−control study, 2019 to 2024
Source: Euro Surveill. 2026 Sep 17;31(37):2500888. doi: 10.2807/1560-7917.ES.2026.31.37.2500888 (PMC13589211; doi:10.2807/1560-7917.ES.2026.31.37.2500888)
Supplement: Supplementary file 3 [file 25-00888_WHITELEY_Supplement3.pdf]

This supplementary material is hosted by Eurosurveillance as supporting information alongside the article 'Factors driving increases in Clostridioides difficile infection rates in England: A national case-control study, 2019 to 2024', on behalf of the authors, who remain responsible for the accuracy and appropriateness of the content. The same standards for ethics, copyright, attributions and permissions as for the article apply. Supplements are not edited by Eurosurveillance and the journal is not responsible for the maintenance of any links or email addresses provided therein.

Antibiotics considered

These are the BNF codes used:

Class	Drug	BNF code
Amoxicillin		0501013B0%
Co-Amoxiclav		0501013K0%
Clindamycin		0501060%
Cephalosporins (1st gen)	Cefadroxil	0501021B0%
	Cefalexin	0501021L0%
	Cefradine	0501021M0%
Fluroquinolones	Ciprofloxacin	0501120L0%
	Levofloxacin	0501120XL0%
	Moxifloxacin	0501120Y0%
	Norfloxacin	0501120Q0%
	Ofloxacin	0501120P0%
Proton Pump Inhibitors		010305%

Note also that for Cephalosporins, we also looked at 2nd and 3rd generation. However, all patients that had 2nd or 3rd gen cephalosporins in the community in the 6 months prior to admission or diagnosis also had 1st generation cephalosporins (39 CDI patients and 64 controls).

Time period sensitivity

A period of 6 months was deemed epidemiologically appropriate time to consider antibiotic effect on the gut. However, to test/ explore this we ran sensitivity analysis considering alternatives of 3, 6, 9 and 12 months. The results can be seen in the figure below. We see that the odds ratio is highest for shorter periods considered. However, correlation was observed even up to 1 year (the maximum we could model). A period of six months to have taken antibiotics before infection/ hospitalisation seemed a reasonable assumption to make.

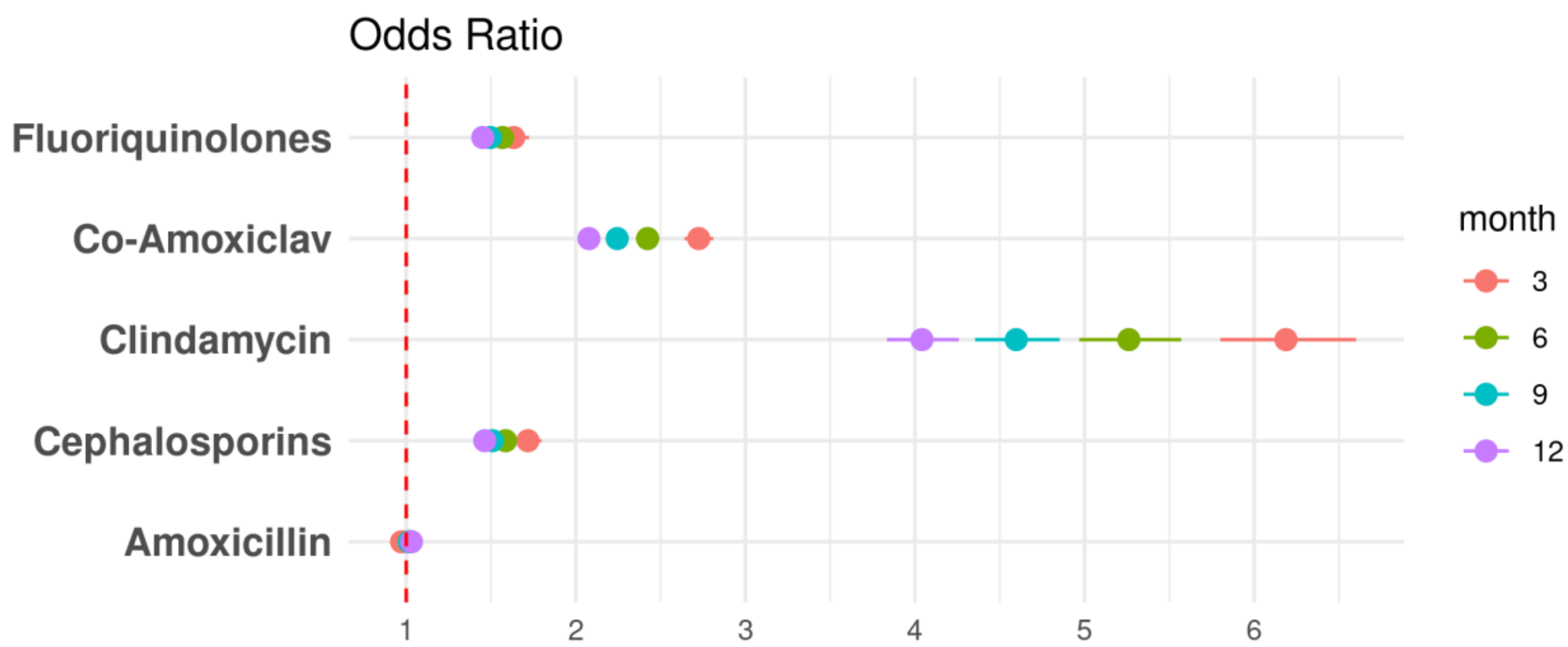
Odds Ratio
Fluoriquinolones
Co-Amoxiclav
Clindamycin
Cephalosporins
Amoxicillin
1
2
3
4
5
6
month
3
6
9
12
