# Supplementary material for: Factors driving increases in Clostridioides difficile infection rates in England: a national case−control study, 2019 to 2024
Source: Euro Surveill. 2026 Sep 17;31(37):2500888. doi: 10.2807/1560-7917.ES.2026.31.37.2500888 (PMC13589211; doi:10.2807/1560-7917.ES.2026.31.37.2500888)
Supplement: Supplementary file 4 [file 25-00888_WHITELEY_Supplement4.pdf]

This supplementary material is hosted by Eurosurveillance as supporting information alongside the article 'Factors driving increases in Clostridioides difficile infection rates in England: A national case-control study, 2019 to 2024', on behalf of the authors, who remain responsible for the accuracy and appropriateness of the content. The same standards for ethics, copyright, attributions and permissions as for the article apply. Supplements are not edited by Eurosurveillance and the journal is not responsible for the maintenance of any links or email addresses provided therein.

Appendix E: Tables corresponding to figures

	All cases			Community Onset			Hospital Onset		
year	Observed	Predicted	Percentage explained	Observed	Predicted	Percentage explained	Observed	Predicted	Percentage explained
2019-20	9273	9273	100.0	5442	5442	100.0	3831	3831	100.0
2020-21	8771	7699	87.8	5359	4493	83.8	3412	3196	93.7
2021-22	10265	9320	90.8	5983	5434	90.8	4282	3904	91.2
2022-23	11012	10257	93.1	6019	5976	99.3	4993	4296	86.0
2023-24	11752	11074	94.2	6794	6437	94.7	4958	4652	93.8

Table E1: Values used for Figure 2, as well as the percentage of observed cases that are explained by the model.

Year	Baseline	Model 1	Model 2	Model 3	Full model	Observed
2019-20	9273	9273	9273	9273	9273	9273
2020-21	7010	7074	7310	7510	7699	8771
2021-22	8767	8765	8972	9135	9320	10265
2022-23	9177	9262	9437	9703	10257	11012
2023-24	9797	9923	10043	10410	11074	11752

Table E2: Values corresponding to Figure 3.
