# Supplementary material for: Factors driving increases in Clostridioides difficile infection rates in England: a national case−control study, 2019 to 2024
Source: Euro Surveill. 2026 Sep 17;31(37):2500888. doi: 10.2807/1560-7917.ES.2026.31.37.2500888 (PMC13589211; doi:10.2807/1560-7917.ES.2026.31.37.2500888)
Supplement: Supplementary file 5 [file 25-00888_WHITELEY_Supplement5.pdf]

This supplementary material is hosted by Eurosurveillance as supporting information alongside the article 'Factors driving increases in Clostridioides difficile infection rates in England: A national case-control study, 2019 to 2024', on behalf of the authors, who remain responsible for the accuracy and appropriateness of the content. The same standards for ethics, copyright, attributions and permissions as for the article apply. Supplements are not edited by Eurosurveillance and the journal is not responsible for the maintenance of any links or email addresses provided therein.

Appendix B: Comparing only those community onset cases close to their hospitalization episode.

In the table below, we can see the odds ratios for all community onset cases and the subset of CO cases that had their CDI infection either 84 days before or after their hospitalization.

Most variables are not statistically significantly different. Unknown ethnicities are different but there is a small data set of these and this may not be reflective of a difference. Age is significantly different but the value is the same to 2 decimal places, as is previous hospitalisation. Renal disease, cancer, comorbidities, co-amoxiclav and clindamycin match the pattern seen in the difference between community and hospital onset cases. If we exclude cases that are clearly community onset, the remaining cases will be more like the hospital onset cases, which is what we see.

Classified as ECDC NORMAL

	OR for CO	OR CO cases within 84 days	P value
(Intercept)	0.00	0.00	0.052
Age	1.027	1.025	0.000
Sex: Male	0.72	0.74	0.224
Ethnicity: Asian	0.74	0.73	0.783
Ethnicity: Black	0.57	0.53	0.444
Ethnicity: Mixed	0.63	0.60	0.686
Ethnicity: Other	0.77	0.80	0.799
Ethnicity: Unknown	1.54	1.20	0.000
Financial Quarter: 2019/20 Q2	1.20	1.18	0.707
Financial Quarter: 2019/20 Q3	1.04	1.03	0.765
Financial Quarter: 2019/20 Q4	1.05	1.01	0.483
Financial Quarter: 2020/21 Q1	1.46	1.39	0.416
Financial Quarter: 2020/21 Q2	1.37	1.32	0.555
Financial Quarter: 2020/21 Q3	1.23	1.20	0.686
Financial Quarter: 2020/21 Q4	1.20	1.14	0.432
Financial Quarter: 2021/22 Q1	1.26	1.24	0.806
Financial Quarter: 2021/22 Q2	1.38	1.36	0.737
Financial Quarter: 2021/22 Q3	1.17	1.13	0.546
Financial Quarter: 2021/22 Q4	1.00	0.96	0.439
Financial Quarter: 2022/23 Q1	1.11	1.09	0.710
Financial Quarter: 2022/23 Q2	1.31	1.25	0.343
Financial Quarter: 2022/23 Q3	1.03	0.96	0.217
Financial Quarter: 2022/23 Q4	0.95	0.91	0.523
Financial Quarter: 2023/24 Q1	1.09	1.07	0.824
Financial Quarter: 2023/24 Q2	1.17	1.13	0.567
Financial Quarter: 2023/24 Q3	1.13	1.10	0.653
Financial Quarter: 2023/24 Q4	1.23	1.16	0.239
Frailty	1.12	1.13	0.047
Renal Disease	1.18	1.35	0.000
Total cips duration	1.01	1.01	0.001
Cancer	0.92	1.08	0.000
Comorbidities	1.10	1.14	0.000
Testing rate	1.01	1.01	0.583
IMD: 1	0.99	1.03	0.270
IMD: 2	1.04	1.09	0.267
IMD: 3	1.02	1.05	0.391
IMD: 4	0.99	0.99	0.839
IMD: 5	0.98	1.00	0.561
IMD: 6	0.97	0.97	0.933
IMD: 7	0.95	0.95	0.863

Classified as ECDC NORMAL

IMD: 8	1.01	1.01	0.924
IMD: 9	1.04	1.04	0.956
IMD: Unknown	0.93	0.98	0.314
Region: East of England	1.01	1.04	0.497
Region: London	0.73	0.76	0.261
Region: North East	0.98	1.01	0.508
Region: North West	1.17	1.10	0.128
Region: South East	0.95	0.94	0.785
Region: South West	1.07	1.04	0.579
Region: West Midlands	0.92	0.93	0.873
Region: Yorkshire and Humber	1.09	1.08	0.823
Amoxicillin	1.11	1.08	0.149
Co-Amoxiclav	3.02	2.81	0.006
Clindamycin	7.19	6.25	0.005
Cephalosporins	1.78	1.76	0.854
Fluoriquinolones	1.77	1.75	0.818
Renal Disease * Total cips duration	1.00	1.00	0.060
Cancer * Total cips duration	1.00	1.00	0.103

Classified as ECDC NORMAL
